# Physiological and ecological effects of increasing temperature on fish production in lakes of Arctic Alaska

**DOI:** 10.1002/ece3.1080

**Published:** 2014-04-22

**Authors:** Michael P Carey, Christian E Zimmerman

**Affiliations:** U.S. Geological Survey, Alaska Science Center4210 University Dr., Anchorage, Alaska, 99508

**Keywords:** Arctic Coastal Plain, climate change, coregonidae, fish bioenergetics, lake food webs

## Abstract

Lake ecosystems in the Arctic are changing rapidly due to climate warming. Lakes are sensitive integrators of climate-induced changes and prominent features across the Arctic landscape, especially in lowland permafrost regions such as the Arctic Coastal Plain of Alaska. Despite many studies on the implications of climate warming, how fish populations will respond to lake changes is uncertain for Arctic ecosystems. Least Cisco (*Coregonus sardinella*) is a bellwether for Arctic lakes as an important consumer and prey resource. To explore the consequences of climate warming, we used a bioenergetics model to simulate changes in Least Cisco production under future climate scenarios for lakes on the Arctic Coastal Plain. First, we used current temperatures to fit Least Cisco consumption to observed annual growth. We then estimated growth, holding food availability, and then feeding rate constant, for future projections of temperature. Projected warmer water temperatures resulted in reduced Least Cisco production, especially for larger size classes, when food availability was held constant. While holding feeding rate constant, production of Least Cisco increased under all future scenarios with progressively more growth in warmer temperatures. Higher variability occurred with longer projections of time mirroring the expanding uncertainty in climate predictions further into the future. In addition to direct temperature effects on Least Cisco growth, we also considered changes in lake ice phenology and prey resources for Least Cisco. A shorter period of ice cover resulted in increased production, similar to warming temperatures. Altering prey quality had a larger effect on fish production in summer than winter and increased relative growth of younger rather than older age classes of Least Cisco. Overall, we predicted increased production of Least Cisco due to climate warming in lakes of Arctic Alaska. Understanding the implications of increased production of Least Cisco to the entire food web will be necessary to predict ecosystem responses in lakes of the Arctic.

## Introduction

Lakes are prominent features across the Arctic landscape, especially in lowland permafrost regions where the landscape comprises thousands of lakes and the lake area fraction (lake area/land area) can exceed 40%, such as on the Arctic Coastal Plain of Alaska (Grosse et al. 2013). In addition to their intrinsic value, lakes are sentinels for Arctic ecosystems as integrators of climate-induced physical changes (Schindler 2009; Williamson et al. 2009). Climate warming is having profound effects on the physical characteristics of freshwater ecosystems in the Arctic (Wrona et al. 2006). Changes to Arctic lakes include warmer water temperatures and shorter periods of ice cover (Rouse et al. 1997; Clilverd et al. 2009; Williamson et al. 2009; Arp et al. 2010; Busch et al. 2012). While these physical transformations have been documented, how aquatic species will respond to these changes is poorly understood in Arctic lakes. Examining the effect of climate-induced changes on fish can provide insights into how Arctic food webs will respond to climate warming (Schindler 2009). Changes in the characteristics of fish populations (e.g., size structure, abundance, and growth rates) have indicated ecosystem changes in other freshwater systems and provided guidance for conservation strategies to mitigate the effect of eutrophication, invasive species, etc., (Sebens 1987; Carey and Mather 2008).

Warmer water temperature is likely the foremost physical change occurring in lakes from climate warming. Water temperature has a myriad of effects on limnological processes and plays an important role in the distribution and life history of fish, along with other aquatic organisms (Magnuson et al. 1997; Casselman 2002; Sharma et al. 2007). In fact, temperature is the main factor directly governing the biology of fish and ultimately determines their production and survival (Christie and Regier 1988; Magnuson et al. 1990; Sharma et al. 2007; Busch et al. 2012). The implications of higher lake temperatures on fish production have not been thoroughly studied for fish populations in lakes on the Arctic Coastal Plain; at this point, most predictions by researchers are based on studies conducted in more temperate systems.

In addition to higher water temperatures, climate warming also reduces the duration of ice cover on Arctic lakes (Arp et al. 2010; Kittel et al. 2011; Shuter et al. 2012). In the future, lake ice cover is expected to be thinner, break-up earlier, freeze-up later, and lead to a longer ice-free season (Rouse et al. 1997). Ice phenology shapes the annual life cycle of all the organisms in freshwater ecosystems in the Arctic including fish. The duration of the open-water period determines the length of the growing season for fish, whereas the period of ice cover is a time of increased mortality from negative energy budgets and potentially low oxygen (Brown et al. 2001; Shuter et al. 2012; Weber et al. 2013). Lakes may become deficient in oxygen from lack of free exchange with the atmosphere and limited photosynthesis due to shortened day length and snow cover (Clilverd et al. 2009). Thus, changes to ice cover will likely have profound effects on fish production in Arctic lakes.

Climate-induced changes to the chemical and physical characteristics of lakes may indirectly influence fish production by altering energy flow through the food web (e.g., Reist et al. 2006b; Wrona et al. 2006; Chételat and Amyot 2009; Kittel et al. 2011; Shuter et al. 2012). For instance, climate change has altered species composition and production of phytoplankton and zooplankton in Arctic and Subarctic lakes (Schindler et al. 2005; Chételat and Amyot 2009; Carter and Schindler 2012). Changes to primary production and the invertebrate community could impact the fish community and other top predators by bottom-up effects through the food web. Indirect effects of climate warming mediated through the food web have the potential to supplement direct temperature effects in governing fish production.

**Figure 1:**
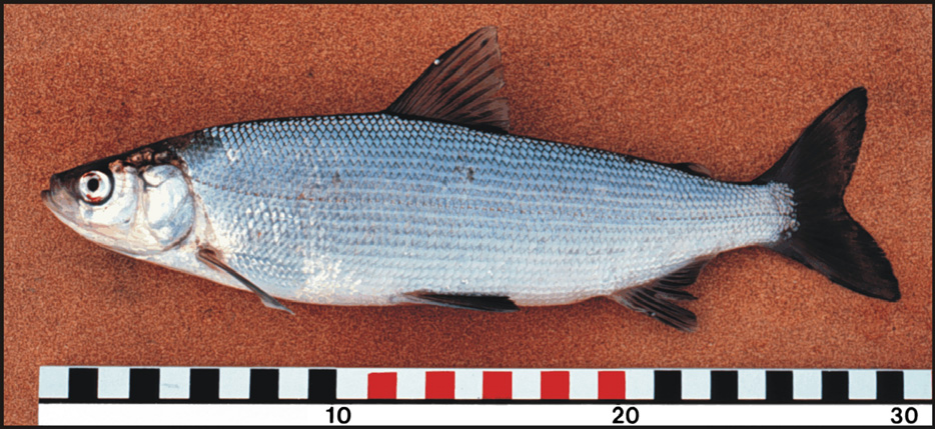
Least Cisco caught in Arctic Alaska (photo credit R. Brown).

Currently, we have limited basic ecological information on fish populations in Arctic systems and need to develop ecological models to guide empirical research and address pressing management decisions. Least Cisco (*Coregonus sardinella;* Fig. 1) may be a bellwether for lakes on the Arctic Coastal Plain as an important consumer foraging mostly on zooplankton in the water column. The importance of fish as consumers on food web properties has been found in other systems (Covich et al. 2004; Worm et al. 2006; Burkepile and Hay 2008; Hargrave 2009; Carey and Wahl 2011a,b). Moreover, fish can have bottom-up effects on food webs by excretion in small, oligotrophic systems (Eby et al. 2006; Hargrave 2006; Abe et al. 2007). Least Cisco is also an important prey resource for top predators, such as northern pike (*Esox lucius*) and loons (*Gavia spp*.). Therefore, changes in growth rates and size structure of Least Cisco could have implications throughout the food web. Least Cisco will likely have strong responses to climate warming as temperatures exceed the optimum temperature of 16.8°C for coregonids (Binkowski and Rudstam 1994). Furthermore, coregonids provide opportunities to the residents of the Arctic Coastal Plain villages for subsistence harvest (Hershey et al. 2006 and references therein).

Climate change effects on temperature could result in different water temperatures, length of ice-free season, and energy flow through the food web, all of which have consequences relevant to Least Cisco and food webs on the Arctic Coastal Plain. To explore these consequences, we used a bioenergetics model to simulate changes in fish growth under different climate scenarios. Bioenergetic models provide a framework to test hypotheses about climate change by linking physiological and ecological factors (Hanson et al. 1997; Kitchell et al. 1977; Beauchamp 2009; Shuter et al. 2012). Moreover, growth rates of individuals have long been used across plant and animal taxa to monitor health and understand ecosystem change (Sebens 1987). First, we use current water temperatures to fit an estimate of consumption with the bioenergetics model to observed growth of Least Cisco. We then estimated annual growth of Least Cisco, holding food availability and feeding rates constant, using projected temperature increases for 2040, 2060, and 2090 from Scenarios Network for Alaska and Arctic Planning (SNAP 2013). Second, we compare temperature increases in the summer (open water; June–September) to increases in the winter (ice-covered; October–May) to determine the relative importance of seasonal changes. Third, we explore the consequences of a shorter period of ice cover independent of an increase in average summer temperatures. Finally, we consider the influence of prey quality on Least Cisco as a means to examine the impact of differences in energy flow resulting from temperature changes.

## Methods

### Study area

The Arctic Coastal Plain of Alaska is a complex landscape of low relief underlain by continuous permafrost and overlain in major areas by a dominant hydrological complex of rivers and interconnected, shallow lakes, and wetlands. The thermokarst process of this ice-dominated landscape creates lakes with a range of surface areas, depths, and types of interconnections (Grosse et al. 2013). Nearly, 40% of the surface area of the Arctic Coastal Plain is covered by lakes that are cold and typically monomictic (Clilverd et al. 2009). Submerged aquatic vegetation is not abundant in these systems and the trophic status is highly oligotrophic (Clilverd et al. 2009; J. Koch, unpubl. data). During the ice-free season, lakes are often well mixed and a strong relationship exists between air and water temperature (Shuter and Post 1990; Livingstone and Lotter 1998; Livingstone and Dokulil 2001; Sharma et al. 2007; Arp et al. 2011). Lakes shallower than ∼2 m generally freeze to the bottom and unfrozen water in deeper lakes can become hypoxic (Clilverd et al. 2009). Thaw-lake depth is controlled by air temperature, winter lake ice thickness, and snow cover (Kittel et al. 2011).

Lakes on the Arctic Coastal Plain contain simple fish communities. The most commonly found species are ninespine stickleback (*Pungitius pungitius*) and Alaska blackfish (*Dallia pectoralis*), whereas widely distributed, but found more often in highly connected systems, are Least Cisco, Broad whitefish (*Coregonus nasus*), and Arctic grayling (*Thymallus arcticus*). Similar to nearby systems in the Brooks Range (Hershey et al. 1999), stream connections and lake depth influence species composition (Haynes et al. 2013). More sporadically found are slimy sculpin (*Cottus cognatus*), Dolly varden (*Salvelinus malma*), rainbow smelt (*Osmerus mordax*), Humpback whitefish (*Coregonus oidschian*), and the top predators Northern pike (*Esox lucius*) and burbot (*Lota lota*; Haynes et al. 2013; S. Laske, unpubl. data; J. Schmutz, unpubl. data).

### Bioenergetics of Least Cisco

We assessed the metabolic response of Least Cisco to predicted changes in temperature using the Wisconsin bioenergetics model (Hanson et al. 1997). This model is a mass/balance equation where consumption (C) equals growth (G) minus the energy cost of respiration (M) and waste (W), C = G + M + W. Growth is determined by somatic growth and gonad production. Metabolism involves respiration, active metabolism, and specific dynamic action, whereas waste includes egestion and excretion. We use the generalized coregonid model (Hanson et al. 1997) with physiological variables (respiration, waste, and growth) parameterized according to Rudstam et al. (1994) and Madenjian et al. (2006). Respiration parameters were specified to Least Cisco (RA = 0.00325) using values from Wohlschag (1957). The respiration parameter in the coregonid model has led to overestimations of consumption in other coregonids (Madenjian et al. 2006). Predator energy density was estimated using the equation from Johnson et al. (1998) for lake herring to account for size differences among age classes. Least Cisco normally mature between 5 and 7 years of age (Mann and McCart 1981; Philo et al. 1993a,b1993b; Moulton et al. 1997; Seigle 2003). We simulated the cost of spawning for fish age-6 and older by estimating a 10% loss of body mass as was found for coregonids in Lake Superior (Johnson et al. 1998). Spawning occurs in early to late fall often during and after freeze-up with females depositing eggs over sand or gravel that are then fertilized by the males (Seigle 2003). We modeled spawning to occur on 1 October just as ice cover was forming on lakes (Arp et al. 2013).

**Figure 2:**
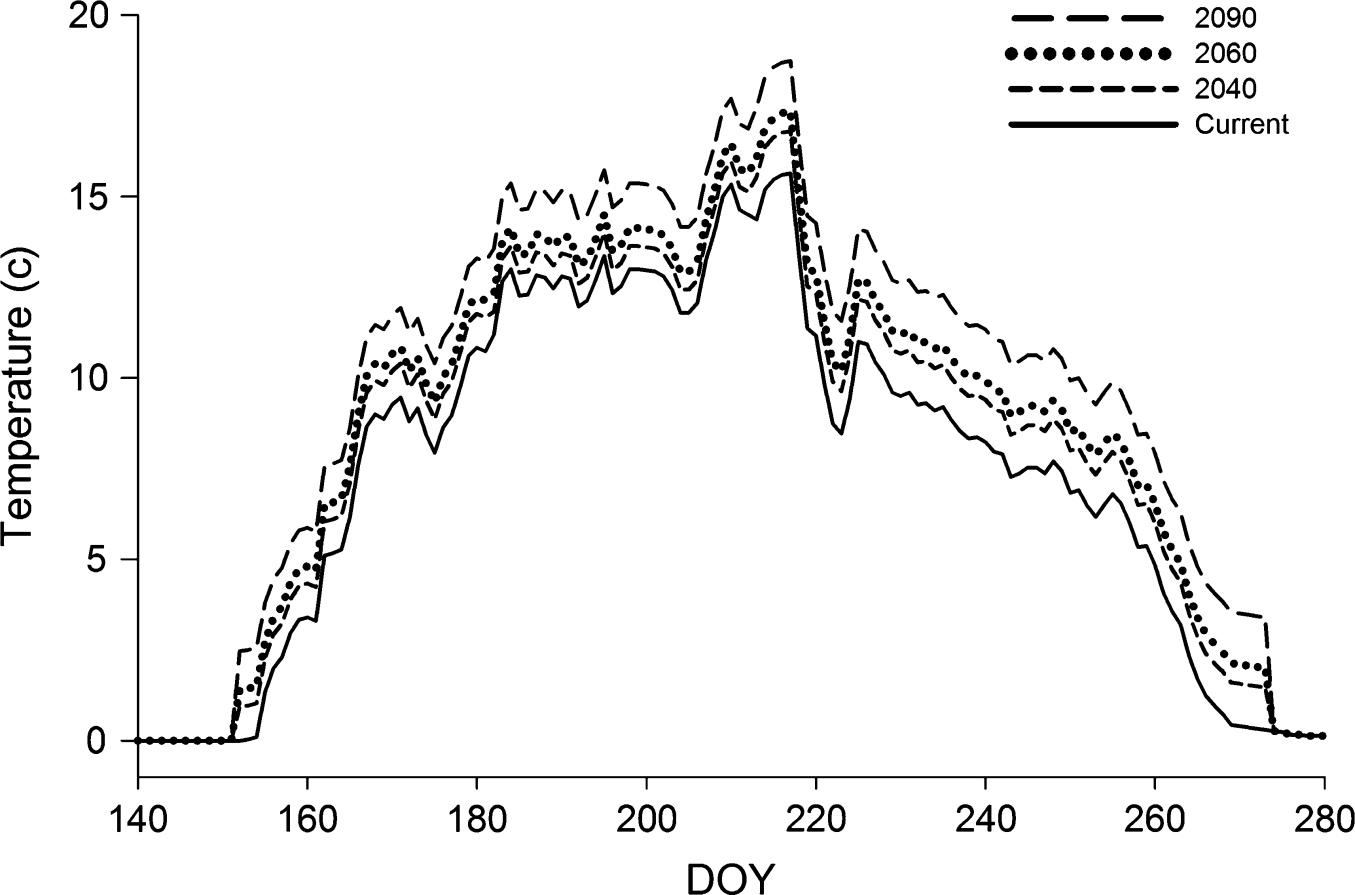
Water temperature data plotted through the ice-free period (day of year, DOY: 152–273) for current conditions (average daily temperature of 2009, 2010, and 2011) and future projections for 2040, 2060, and 2090. Future predictions are calculated as current water temperature plus the predicted increase for air temperature in Atqasuk, AK by the Scenarios Network for Alaska and Arctic Planning (SNAP 2013). We incorporate SNAP projections based on emission scenario B1, A1B, and A2 and averaged values by day for each emission scenario.

To parameterize the Least Cisco model to lakes on the Arctic Coastal Plain, we assumed Least Cisco feed primarily in the water column and their diet remains constant throughout the year. In the model, diets were evenly divided among expected prey groups of bosmina (1674 J/g wet mass), copepods (2792 J/g wet mass), other cladocerans (2514 J/g wet mass), diptera (1763 J/g wet mass), and leptodora (867 J/g wet mass) using previously published values for prey energy density (Hanson et al. 1997; Schindler and Eby 1997). Diet items were divided evenly as we currently lack diet data for Least Cisco on the Arctic Coastal Plain. We assumed that access to prey is accounted for in the observed growth used to fit the model (e.g., Griffiths and Schindler 2012). Next, we modeled consumption based on temperature and growth data from the region. To represent current temperatures throughout the year, daily temperatures were averaged from 2009, 2010, and 2011 from Bill's Creek (Lat. 70.236°N; Long. 151.274°W) in the Fish Creek Watershed, National Petroleum Preserve – Alaska on the Arctic Coastal Plain (Whitman et al. 2011; Fig. 2). Temperatures were measured at a deep pool of this beaded stream with depths similar to the very shallow lakes in the region providing a suitable representation of water temperature. We estimated annual growth of Least Cisco for multiple age classes (Age-2–Age-20) using a von Bertalanffy growth function (length [1 − e^−0.16(age)^]; Seigle 2003). Von Bertalanffy growth curves have been used to represent coregonid biomass in the Laurentian Great Lakes (Rudstam et al. 1994; Johnson et al. 1998) and using a von Bertalanffy growth function in conjunction with a bioenergetics model has been insightful for other fish species (Essington et al. 2001). Weight (g) was estimated from length weight regressions by combining predictions based on field data from Ikrouvik Lake, Alaska and near Point Barrow, Alaska (Carlander 1969). Annual growth was estimated by biomass differences between age classes (Table 1). Using these growth estimates, we calculated the proportion of maximum consumption (pCmax), as a measure of feeding rate, and annual ration (food availability) needed to produce the observed growth of fish based on diet composition, prey energy density, and daily temperature throughout the year for each age class of Least Cisco (Table 1). Annual ration (percent biomass of fish) was calculated as the average daily ration (g/g/d) for each age class (Hanson et al. 1997; Griffiths and Schindler 2012).

**Table 1:** Inputs to the bioenergetics model for fitting Least Cisco consumption to annual growth during current water temperatures using values averaged from the literature for prey energy density (J/g wet mass). Model inputs that varied for each age class are initial mass (g), final mass (g), and predator energy density (J/g wet mass). The model simulations were run for 365 days to determine the proportion of maximum consumption (pCmax) for each age class

Age (years)	Initial mass (g)	Final mass (g)	Predator energy density (J/g)
2	11	30	4678
3	30	59	4906
4	59	95	5246
5	95	136	5674
6	136	179	6158
7	179	223	6673
8	223	265	7195
9	265	306	7693
10	306	344	8179
11	344	379	8630
12	379	410	9045
13	410	438	9413
14	438	463	9745
15	463	486	10,041
16	486	505	10,314
17	505	522	10,478
18	522	537	10,478
19	537	550	10,478
20	550	561	10,478

### Climate predictions

We estimated fish growth (G = C – [M + W]) for different climate scenarios by holding annual ration and pCmax constant at the level determined for current conditions. By holding annual ration and pCmax constant, we can predict growth for future temperature scenarios. Holding feeding rate and food availability constant has been useful in projecting growth differences with climate change predictions in other systems (e.g., Griffiths and Schindler 2012). Climate scenarios were taken from Scenarios Network for Alaska and Arctic Planning (SNAP 2013) projections that are based on three emission scenarios (A1, A1B, and A2). For each emission scenario, monthly temperature projections are made for 2040, 2060, and 2090. SNAP projections predict air temperature; however, there is a strong relationship between water and air temperature in these systems during the ice-free periods (Shuter and Post 1990; Livingstone and Lotter 1998; Livingstone and Dokulil 2001; Sharma et al. 2007). For example, mean July air temperature was the most important variable in predicting maximum lake surface-water temperatures yielding a partial *R*^2^ = 0.70 (Sharma et al. 2007). Thus, we forecast future water temperature regimes by increasing the daily value of the current water temperature by the amount predicted for air temperature during the ice-free period (Fig. 2). Griffiths and Schindler (2012) used a similar approach of projecting future water temperatures by adding predicted change in temperature to observed values through hydrodynamics model. The ice-free period was expected to last from 1 June until 30 September based on data from Lake Teshepuk (Arp et al. 2013).

We modeled changes in winter temperature, duration of the ice cover, and energy flow through the food web–holding pCmax constant in the bioenergetics simulation. Winter is defined as the period of ice cover for a water body (Shuter et al. 2012). We explored changes in winter temperature, although temperatures under the ice will not be directly influenced by increasing air temperature (Griffiths et al. 2011; Griffiths and Schindler 2012). Rather, temperature changes could be due to changing snow depths, different levels of light penetration, or changing hydrologic inputs (Ling and Zhang 2003; Kittel et al. 2011). Due to the uncertainty in forecasting winter conditions, we compared a 1°C increase in daily temperature during the ice-covered period (winter, October–May) to a 1°C increase in daily temperature during the summer. The temperature increase in the summer was for 122 days and the winter for 243 days. We explore the influence of a short period of ice cover by changing 10 or 20 days of ice-covered to ice-free without changing the range of summer temperatures. Increasing the number of ice-free days by 10 or 20 days is within the expected range of increasing ice-free days for the region (Arp et al. 2013). The number of days with ice cover was reduced from the end of May (5 or 10 days) and the beginning of October (5 or 10 days each). We explored the effects of food quality on Least Cisco by altering prey quality. Using the range of prey energy density found in the literature (copepods: 1900–3684 J/g wet mass; other cladocerans: 2281–2746 J/g wet mass; Diptera 1047–2478 J/G wet mass), we reduced the value to the low end of the range found to mimic harsher conditions and increased the prey energy density to the high end of the range to depict more productive conditions. We altered prey quality by changing the value of the prey energy density in the summer and winter separately.

### Growth

Growth of Least Cisco was compared between current conditions and climate change scenarios using the amount of annually accumulated biomass, daily weight accumulated, final weight, and relative weight. Daily weight accumulation is measured as today's mass minus yesterday's mass (g_*t*_ − g_*t*−1_; Hanson et al. 1997). Relative growth ([final mass − initial mass]/initial mass) was calculated to standardize between age classes.

## Results

### Fitting pCmax under current conditions

**Figure 3:**
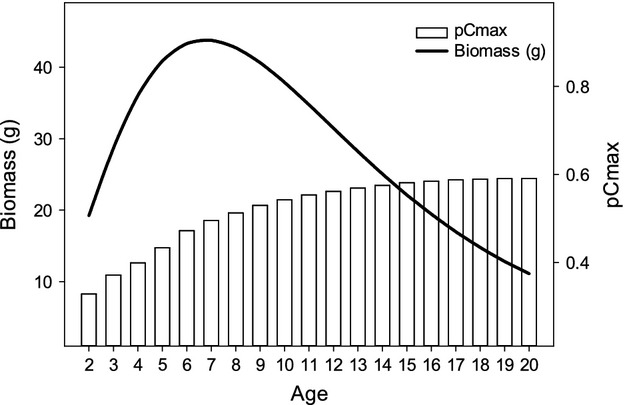
Biomass accumulated for different age classes of Least Cisco and the proportion of maximum consumption (pCmax) estimated for the annual growth of Least Cisco under current temperatures.

**Figure 4:**
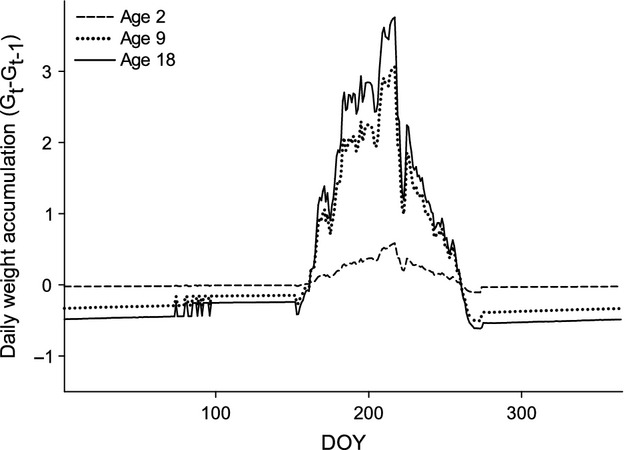
Daily weight accumulation (today's mass minus yesterday's mass) of Least Cisco plotted throughout a year. Age classes of Age-2, 9, and 18 demonstrate seasonal patterns with current temperature conditions.

The proportion of maximum consumption (pCmax) estimated for growth under current temperatures ranged from 0.33 to 0.59 across age classes of Least Cisco (Fig. 3). The pCmax estimates of older age classes are higher than younger age classes despite lower accumulation of biomass each year in older fish. Peak biomass accumulation occurs at age 8 and declines thereafter with older fish. Older age classes of fish have higher estimates of pCmax due to higher predator energy densities for larger fish and the cost of spawning. Daily weight accumulation (G_*t*_ − G_*t*−1_) of Least Cisco indicates growth only occurs during the summer under current temperatures for all age classes (Fig. 4). Biomass accumulation peaks in late July and early August, and there is a loss of biomass during the winter due to cold water temperatures. The seasonal pattern is more evident with larger fish as the older age classes gain more biomass in the summer and lose more biomass in the winter. Despite the strong seasonal pattern, all age classes have positive annual growth with current temperatures.

### SNAP scenarios with constant pCmax

**Figure 5:**
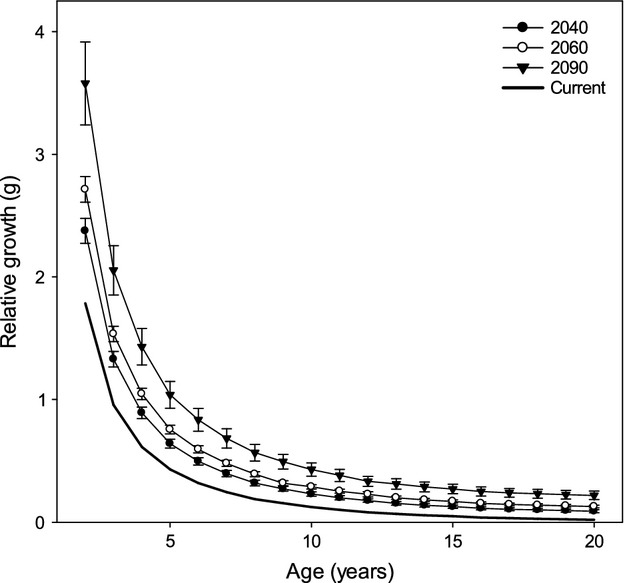
The relative growth estimates ([final biomass − initial biomass]/initial biomass) of Least Cisco are plotted across age classes for current temperatures and for the Scenarios Network for Alaska and Arctic Planning (SNAP 2013) projected temperatures in 2040, 2060, and 2090. The average value (±SD) of biomass is calculated across the three SNAP scenarios (B1, A1B, and A2). Growth estimates are made holding constant the proportion of maximum consumption (pCmax) fitted under current conditions (see Table 1) for projections in 2040, 2060, and 2090.

**Figure 6:**
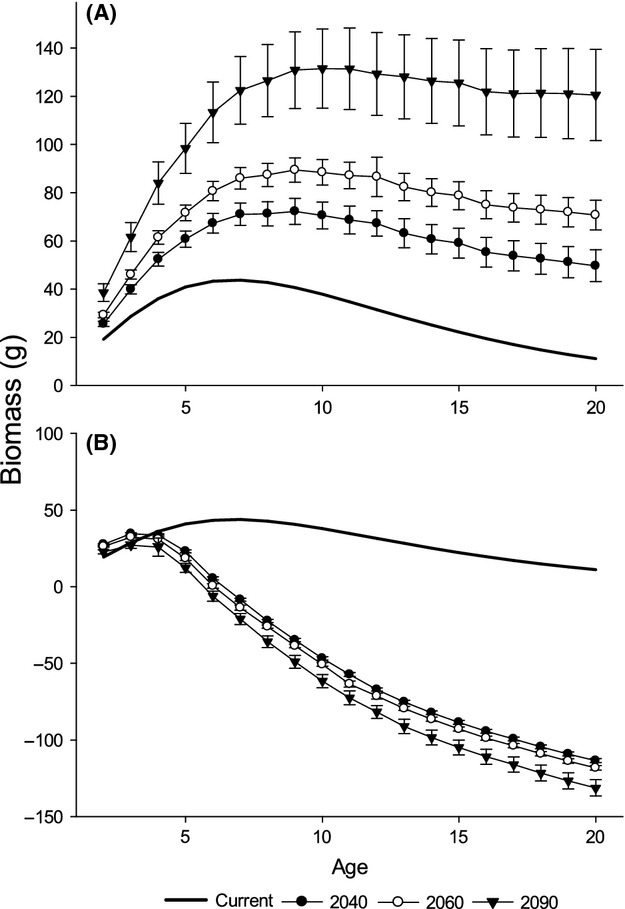
The amount of biomass accumulated annually by each age class of Least Cisco estimated by the bioenergetics model for current temperatures and for the Scenarios Network for Alaska and Arctic Planning (SNAP 2013) projected temperatures in 2040, 2060, and 2090. The average value (±SD) of biomass is calculated across the three SNAP scenarios (B1, A1B, and A2). Biomass estimates are made holding constant (A) the proportion of maximum consumption (pCmax) or (B) the annual ration (percent biomass of fish) for projections in 2040, 2060, and 2090.

Relative growth (g) and accumulated biomass (g) of Least Cisco increased under all future scenarios of warmer temperatures with a constant pCmax (Figs. 5, 6A). Moreover, both measures of production were progressively higher for each warmer temperature projection in 2040, 2060, and 2090 from the baseline of current conditions. Higher variability in estimates of production occurred with time and mirrored the widening divergence between the SNAP projections for warmer temperatures with longer time intervals.

Age classes of Least Cisco responded differently to increasing temperatures. Relative growth shows an exponential decrease from younger to older age classes for all the climate scenarios produced by the SNAP projections (Fig. 5). Younger age classes have the highest relative growth and the most variable estimates. The annual accumulation of biomass peaks at age-7 for current conditions and age-9 for the future climate scenarios (Fig. 6A). In addition to the peak biomass accumulation occur at an older age class with increasing temperature predictions, the older size classes accumulate as much biomass as the middle age classes under the largest temperature increase in 2090.

### SNAP scenarios with constant ration

The annual ration estimated for growth under the current temperatures ranged from 5.61% to 3.18% (percent biomass of fish; Hanson et al. 1997) across age classes. The estimates of annual ration decreased from smaller to larger fish. Holding the annual ration constant for each age class, accumulated biomass (g) of Least Cisco increased for age-2 fish under all future scenarios of warmer temperatures (Fig. 6B). Age-3 fish accumulated more biomass than current conditions in the 2040 and 2060 projections. All other age classes accumulated less biomass than under current conditions for all future scenarios. In fact, age-6 and older age classes are predicted to lose biomass throughout the year under the temperature scenario of 2090 and age-7 and older age classes are estimated to lose biomass under the 2040 and 2060 temperature scenarios.

### Winter scenarios with constant pCmax

**Figure 7:**
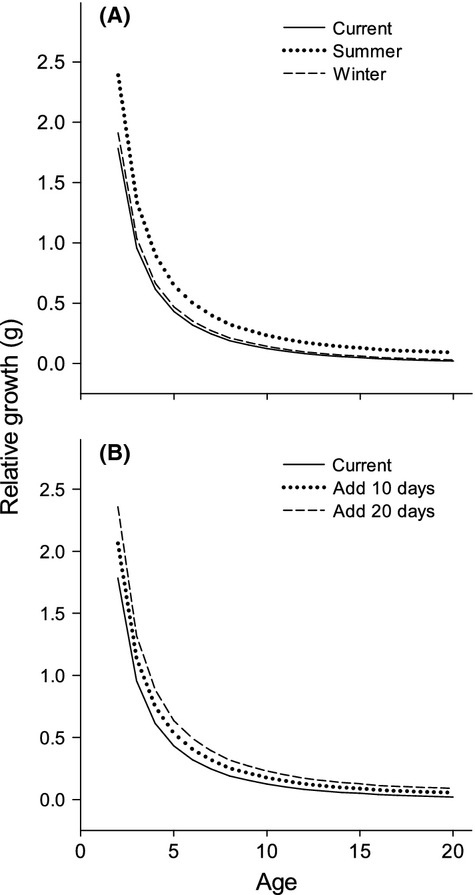
The relative growth estimates ([final biomass − initial biomass]/initial biomass) of Least Cisco are plotted across age classes for (A) warmer water temperature (+1°C) during ice cover or warmer temperatures during open water. (B) Relative growth estimates are plotted for shorter periods of ice cover by adding 10 open-water days or adding 20 open-water days.

Reducing the duration of winter conditions with warmer temperatures and less ice cover increased the relative growth of Least Cisco, but did not change the relationship across age classes. Increasing daily temperature in the winter by 1°C was a minor influence on the annual, relative growth of Least Cisco (Fig. 7A), although less biomass was lost during the ice-covered period. In contrast to the minor effect of changing winter temperature, a 1°C increase in summer created substantially higher production of fish biomass than changes in winter temperatures. Reducing the number of days with ice cover also increased relative growth for all age classes (Fig. 7B). Higher relative growth occurred as the ice-cover period was reduced by adding open-water days and growth continued to increase with a longer open-water period. Overall, increasing the length of the open-water period or the temperatures during the open-water period had a more substantial effect than changing winter conditions.

### Food web effects with constant pCmax

**Figure 8:**
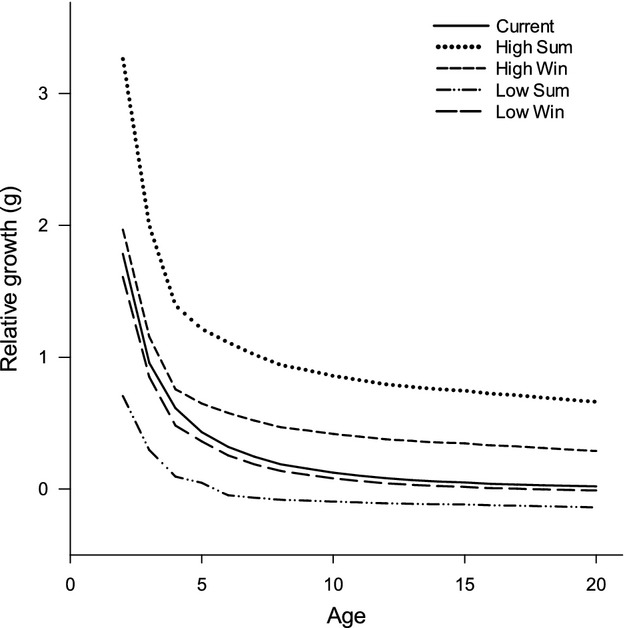
The relative growth estimates ([final biomass − initial biomass]/initial biomass) of Least Cisco are plotted across age classes for differences in prey energy during the summer and winter. Growth estimates are made holding constant the proportion of maximum consumption (pCmax) fitted under current conditions Table 1.

Indirect effects of climate change mediated through prey quality altered production of Least Cisco. Higher prey energy density increased the relative growth of Least Cisco, while lowering prey energy density reduced Least Cisco production (Fig. 8). Increasing or decreasing prey energy density in the summer had a more substantial effect than changes in the winter and younger age classes of Least Cisco responded more than older age classes. For example, lower prey energy density in winter had less of an effect on older age classes as Least Cisco age-14 or older changed by less than 0.02 g of relative weight; whereas age-2 relative growth changed by 1.61 g.

## Discussion

Our model simulations provided a relative comparison of Least Cisco production under different scenarios of climate warming. Bioenergetics models are well suited to explore relative comparisons of different environmental conditions (Chipps and Wahl 2008). Under current temperatures, fitting the bioenergetics model to growth of Least Cisco produced values for the proportion of maximum consumption (pCmax) that ranged from 33% to 59% across age classes. These estimates of pCmax (feeding rate) are within the range observed for coregonids in other systems (12.8–61.5% pCmax; Rudstam et al. 1994). Our estimates of total consumption (g) for Least Cisco also fit with observed values for coregonids found in the Laurentian Great Lakes (Johnson et al. 1998; Madenjian et al. 2006). Differences in the pCmax across age classes may occur in these small lakes due in part to the homogeneous temperatures throughout the water column that limit different-sized fish from finding their optimum value. In addition, higher pCmax values for larger fish indicate their physiological differences including higher predator energy density and the costs of reproduction (Rudstam et al. 1994). Higher pCmax with older, larger fish is a trend found for bloater (*Coregonus hoyi*) in Lake Michigan (Rudstam et al. 1994).

While maintaining feeding rates, all age classes of Least Cisco accumulated biomass annually, peaking at age-7 and tapering off with older age classes. Daily growth had a seasonal pattern. Least Cisco production was high with warm, summer temperatures, whereas biomass decreased in cold, winter temperatures. Our model predictions fit with the expectation that fish lose biomass during the winter in Arctic and Subarctic lakes (Shuter et al. 2012). Winter energy depletion is particularly acute for early life stages, as weight-specific capacity to store energy decreases with smaller body size and energy expenditure increase (Shuter et al. 2012 and references therein). Nevertheless, coregonids have very high feeding rates and grow well even at temperatures substantially lower than their optimum temperature (Binkowski and Rudstam 1994 and references therein). The high potential feeding rates allow coregonids to grow during winter in the Great Lakes (Binkowski and Rudstam 1994). Coregonids in Lake Michigan are able to remain in similar temperatures year round due to deep, cold water and, therefore, have small seasonal changes in consumption (Rudstam et al. 1994). Digestion rates determine feeding rate more than capture efficiency in such cold temperatures. Temperatures in lakes on the Arctic Coastal Plain are much more dynamic reaching lows that reduce Least Cisco growth and highs in which capture efficiency is likely important in determining production.

Using the bioenergetics framework, we predicted climate warming will dramatically impact Least Cisco production in lakes on the Arctic Coastal Plain, similar to forecasts for fish in other high-latitude systems (e.g., Reist et al. 2006b; Griffiths and Schindler 2012). Our growth projections indicate that warmer lake temperatures during the summer will increase growth for all age classes of Least Cisco when feeding rates are maintained into the future. This result is consistent with the pattern of higher specific maximum consumption rates and higher growth as temperatures rise toward the optimum temperature of 16.8°C for coregonids (Binkowski and Rudstam 1994). Not until the prediction for 2090, did temperatures approach values more than a few degrees above the optimum temperature. The current projections of temperature certainly do not reach levels that are likely to cause physiological stress, as the lethal temperature for bloater and cisco is near 26°C (Rudstam et al. 1994). In other regions, climate warming projections predict negative effects on coregonids, such as Vendace (*Coregonus albula*) and Cisco (*Coregonus artedii*), by reducing suitable habitat with warmer temperatures (Elliot and Bell 2011; Kumar et al. 2013). We also found variability in fish production expanding with the length of time forecasted due to the widening disparity of the different SNAP predictions for temperature further into the future. Variability among SNAP predictions of temperature stems from differences in expectations about economic development and assumptions in the general circulation models used to predict temperatures (Sharma et al. 2007).

While the bioenergetics model predicts increased growth with warmer temperatures for all age classes of Least Cisco when maintain feeding rates, we found higher relative growth for younger versus older fish. The youngest age class had the largest percent increase in mass from warmer temperatures in the summer. Reaching a larger size predisposes age-0 fish to higher over-winter survival and may provide a size refuge reducing predation risk (Shuter et al. 1980; Miller et al. 1988; Reist et al. 2006b; Rennie et al. 2010). Size classes responding differently suggests fish species will show a diversity of responses to increasing water temperatures as thermal shifts affect development times and bioenergetics requirements of different species and life stages (Reist et al. 2006a,b2006b; Beauchamp 2009; Kittel et al. 2011). Ultimately, the effects of warmer temperatures will propagate through life stages altering the age of peak biomass accumulation and possibly age of reproduction. An important next step is to consider the entire population structure of Least Cisco and consider how changes in fish production will alter food web interactions.

Climate change influences are also substantial in the winter. In fact, for air temperature, projected winter temperature increases are of a greater magnitude than summer temperature increases on the Arctic Coastal Plain (Griffiths et al. 2011; Griffiths and Schindler 2012; SNAP 2013). Assuming a linear relationship between air and water temperature in the winter would be misleading due to ice-cover (Griffiths et al. 2011; Griffiths and Schindler 2012). Thus, we chose to model a small increase in temperature to explore the implications of warmer water during the winter months. Warmer water temperatures in the winter increased fish growth, but the positive effect was small relative to changes in summer temperature. The growth increases for warmer temperatures in the winter are much less than increasing the water temperature in summer by 1°C or by the SNAP projections. Indeed, the winter period is a longer amount of time, but a daily change of 1°C does not have a large effect on Least Cisco growth and the harsh conditions dominate. A current area of research is trying to understand how climate change will influence lakes during the winter including temperature, snow cover, and ice thickness (Kirillin et al. 2012; Shuter et al. 2012). Low oxygen under ice cover also needs to be addressed especially as water temperatures in winter are warming and preceded by greater summer production making low dissolved oxygen a major challenge for overwintering fish (Brown et al. 2001; Shuter et al. 2012; Weber et al. 2013). Models of Least Cisco growth should be updated once predictions are refined on the physical changes of lakes during the winter.

Climate warming will also alter the phenology of Arctic lakes, in particular the duration of open water. Predicting changes in ice cover is challenging especially for the timing of the ice-in period (Arp et al. 2010; Kittel et al. 2011). Our scenario of altering the duration of ice cover without altering the range of temperatures had a substantial effect on annual production of fish biomass. Adding 20 days in the middle of the open-water period led to growth increases similar to temperature increases predicted for 2040 by the SNAP projections. Temperature and length of the growing season have been previously related to fish growth in Alaska (Brylinski and Mann 1973; Edmundson and Mazumder 2001; Schindler et al. 2005; Rich et al. 2009). A better understanding of how changes in water temperature interact with the phenology of lakes will improve our ability to predict Least Cisco responses to climate warming.

In conjunction with direct effects of changing environmental conditions, climate change may also indirectly affect Least Cisco by altering food web dynamics. Our simulations found prey quality has as much of an effect as water temperature on Least Cisco production. For instance, raising prey energy density in the summer increased growth by a similar amount as warmer temperatures in 2090. A similar effect was found for coastal cut-throat trout (*Oncorhynchus clarki clarki*) in headwater streams of southern British Columbia, Canada in which their growth rates were more sensitive to prey quality than temperature (Leach et al. 2012). The effect of changing prey energy density was not consistent across seasons. Changing prey energy density in the summer produced a more substantial change in Least Cisco growth than a change in prey quality over the winter, despite the longer duration of winter. Yet, the importance of prey quality during the primary growing season fits with the theory of bioenergetics in that if food levels increase, fishes are expected to exhibit greater growth rates at higher temperatures (Magnuson et al. 1997; Brandt et al. 2002; Lehman 2002; Beauchamp 2009). Changes in prey resources can also ameliorate metabolic costs of higher temperature as was found for juvenile sockeye salmon consuming zooplankton in lakes of southwestern Alaska (Griffiths and Schindler 2012). Similar to the effect of temperature, the largest effect from changing prey energy density, whether increasing or decreasing, was on the younger age classes when comparing relative growth (Beauchamp 2009). Prey quality having a larger effect on younger age classes also fits with the theory of bioenergetics as these smaller fish have faster growth rates. In addition, the effect of changing energy density of prey suggests if composition changed in favor of higher or lower energy density species a similar effect would occur as if the current prey became more or less energy dense. Overall, our models confirm the importance of considering how the rest of the food web responds to climate change, as food web dynamics will have strong, indirect impacts on fish production.

If production in lower trophic levels of the food web remains constant despite climate warming, we predicted less annual production for Least Cisco with unchanged food availability in warmer temperatures. The warmer temperatures of the SNAP projections are metabolically costly for Least Cisco to grow at the current level of food availability. A similar decrease was predicted for Lake Trout in larger Arctic Lakes, where primary production is not expected to increase with climate warming (McDonald et al. 1996). Nevertheless, we anticipate increasing prey availability due to warmer temperatures and increased nutrient availability for lakes on the Arctic Coastal Plain as predicted for planktonic production in ponds and small lakes in Subarctic and Arctic Canada, Alaska, northern Scandinavia, and Greenland (Rautio et al. 2011). Whether increase, decreasing, or remaining constant, how lower trophic levels respond to climate warming will be a determinant of Least Cisco production in the future.

Using the generalized coregonid model for comparing differences in Least Cisco growth on the Arctic Coastal Plain provides relative comparisons of future climate projections and identifies knowledge gaps. We used a value for the respiration parameter (Wohlschag 1957) that likely overestimates respiration rates (Rudstam et al. 1994). We used this value as it results in a conservative estimate of growth and is a value determined for Least Cisco in the Arctic. The generalized coregonid model was originally developed for bloater in Lake Michigan (Hanson et al. 1997) and refining the physiological parameters for Least Cisco in the Arctic freshwater would benefit future research and ultimately inform management decisions for Least Cisco. Refinements of the physiological parameters in the bioenergetics model have improved predictions for lake whitefish (*Coregonus clupeaformis*) in Lake Michigan (Madenjian et al. 2006). In addition, even within a species, regional differences in species physiology can have an important influence on the accuracy of parameter estimates (Chipps and Wahl 2008). Regional differences are likely relevant for Least Cisco as individuals have different life-history patterns and behaviors. Individuals may reside entirely in freshwater, while others are amphidromous (Seigle 2003 and references therein). Understanding these life-history variations is important in determining energy sources, growth rates, and future predictions of Least Cisco performance.

The empirical data available on the physical and biological components of Arctic freshwater systems are increasing; however, our only option was to compile data from a variety of sources and regions. With a spatially focused assessment of Least Cisco, a more robust model could be built as large differences occur across the landscape. For example, climate warming in the winter is expected to affect coastal areas more than inland areas due to the influence of a longer marine ice-free period. In contrast, during the summer, the projected temperature increases are of a lesser magnitude on the coast and more pronounced in inland areas. Within a region, systematic differences in lake size and morphometry can generate differences in both ice phenology and summer water temperatures despite common climate conditions (Shuter et al. 2012). Overall, collecting data in the same systems on Least Cisco diets and the prey energy of their diet items would strengthen model predictions, as would a better understanding of the importance and changes in connectivity among lakes, and movement patterns of Least Cisco between lakes. Linking physical and biological processes to forecast future fish performance will direct research for these aquatic food webs and inform adaptive management strategies.
